# Supplementary material for: Multiple Clonostachys rosea UDP-Glycosyltransferases Contribute to the Production of 15-Acetyl-Deoxynivalenol-3-O-Glycoside When Confronted with Fusarium graminearum
Source: J Fungi (Basel). 2023 Jul 2;9(7):723. doi: 10.3390/jof9070723 (PMC10381798; doi:10.3390/jof9070723)

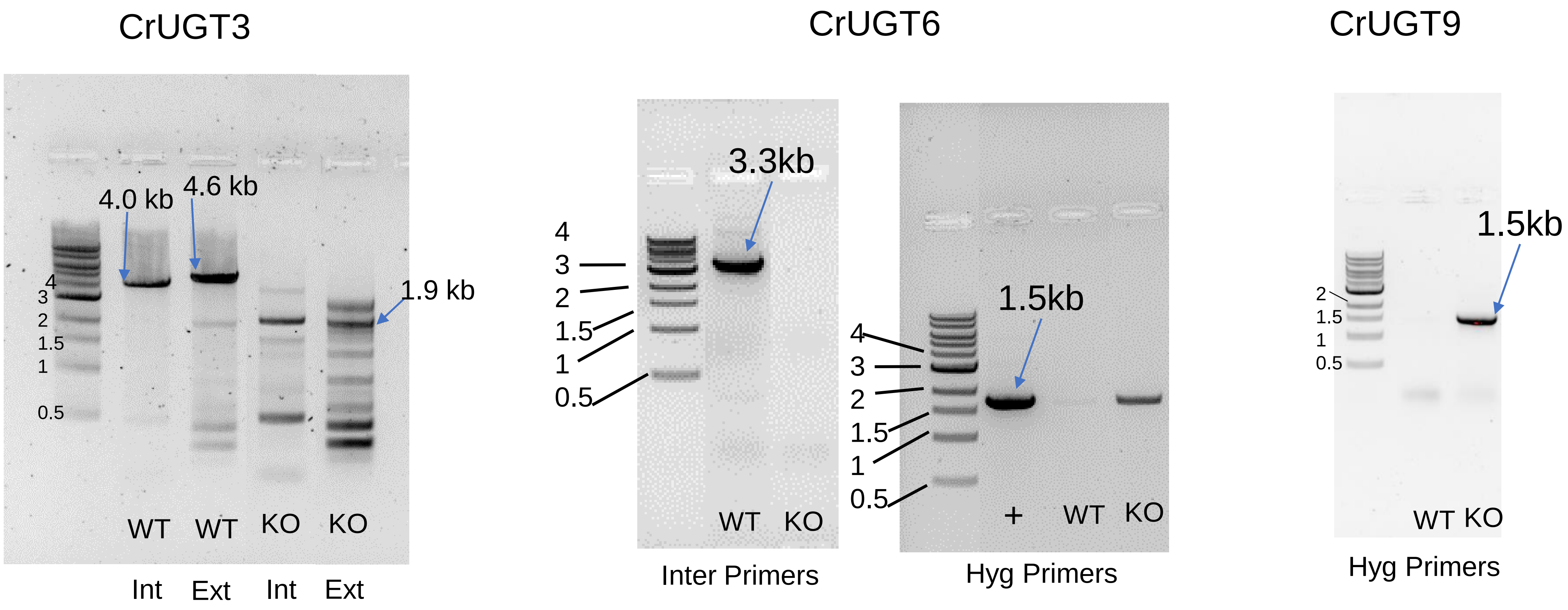

Supplemental Figure S1A: PCR validation of the Crispr/Cas9 knockouts (KOs) of CrUGTs with highest reduction sin the accumulation of 15-ADON-3G. CrUGT3 was tested by PCR using primers internal (Int) to the CrUGT3 gene that are expected to yield a band at 4.0kb for WT, as well as primers external (Ext) to the CrUGT3 gene that should yield a band at 4.6kb for WT and 1.9kb if Hygromycin has replaced CrUGT3. CrUGT6 was tested by using primers internal (Int) to CrUGT6 that are expected to yield a band at 3.3 kb for WT, as well as primers internal to the hygromycin B gene (Hyg) which should yield a band at 1.5 kb is hygromycin is present. A positive control for this hygromycin primer set is included based on PCR amplification from a known plasmid containing the gene. CrUGT9 was only tested by PCR using the Hyg primers.

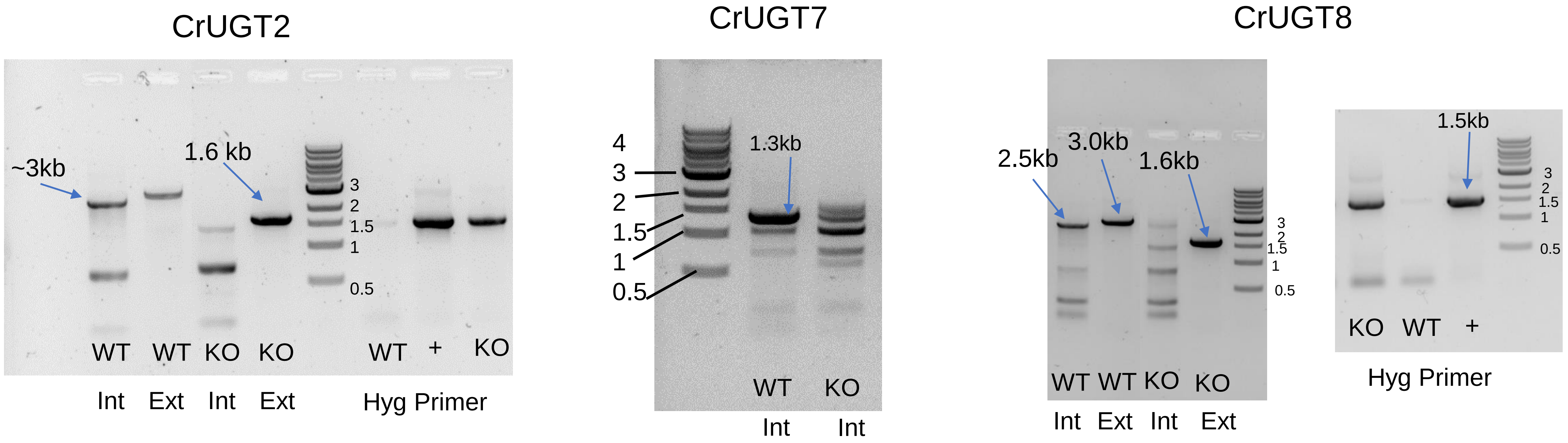

Supplemental Figure S1B: PCR validation of the Crispr/Cas9 knockout strains (KOs) for other CrUGTs. CrUGT2 KO line was tested by PCR using primers internal (Int) to the CrUGT2 gene that are expected to yield a band at 3.0kb for WT, as well as primers external (Ext) to the CrUGT2 gene that should yield a band at 3.5kb for WT and 1.6kb if Hygromycin has replaced CrUGT2. Finally the strain was tested for the presence of the the hygromycin gene using the Hyg primers that should yield a band at 1.5 kb. A positive control for this hygromycin primer set is included based on PCR amplification from a known plasmid containing the gene. CrUGT7 KO line was tested by using primers internal (Int) to CrUGT7 that are expected to yield a band at 1.3 kb for WT. Finally CrUGT8 was tested using primers that were internal (Int) to the CrUGT8 gene that are expected to yield a band at 2.5kb for WT, as well as primers external (Ext) to the CrUGT8 gene that should yield a band at 3.0kb for WT and 1.6kb if hygromycin has replaced CrUGT8. This strain was also tested using primers internal to hygromycin B (Hyg) that should yield a band a 1.5kb if hygromycin has been incorporated. A positive control for this hygromycin primer set is included based on PCR amplification from a known plasmid containing the gene.

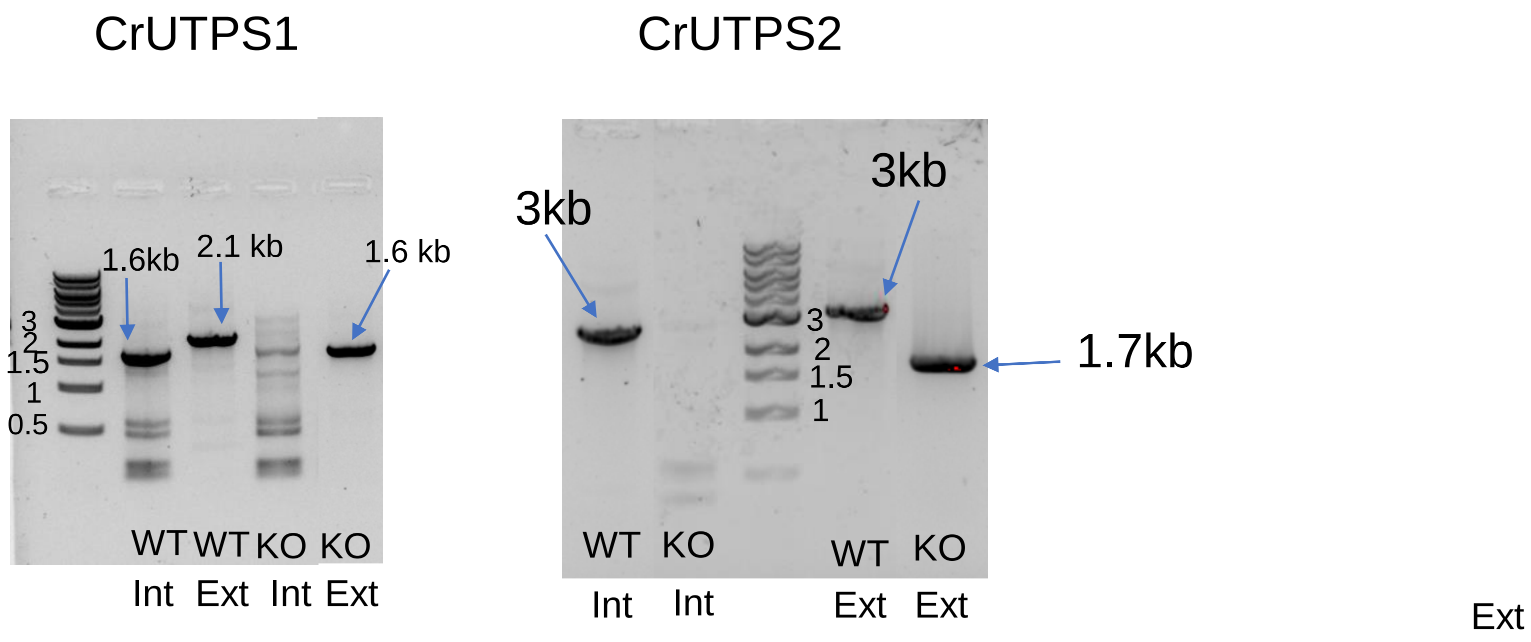

Supplemental Figure S1C: PCR validation of the Crispr/Cas9 knockouts (KOs) of CrUTPSs. CrUTPS1 was tested by PCR using primers internal (Int) to the CrUGT3 gene that are expected to yield a band at 1.6kb for WT, as well as primers external (Ext) to the CrUTPS1 gene that should yield a band at 2.1kb for WT and 1.6kb if Hygromycin has replaced CrUTPS1. CrUTPS2 was tested by using primers internal (Int) to CrUTPS2 that are expected to yield a band at 3.0 kb for WT, primers external (Ext) to the CrUTPS2 gene that should yield a band at 3.0kb for WT and 1.7kb if Hygromycin has replaced CrUTPS2.

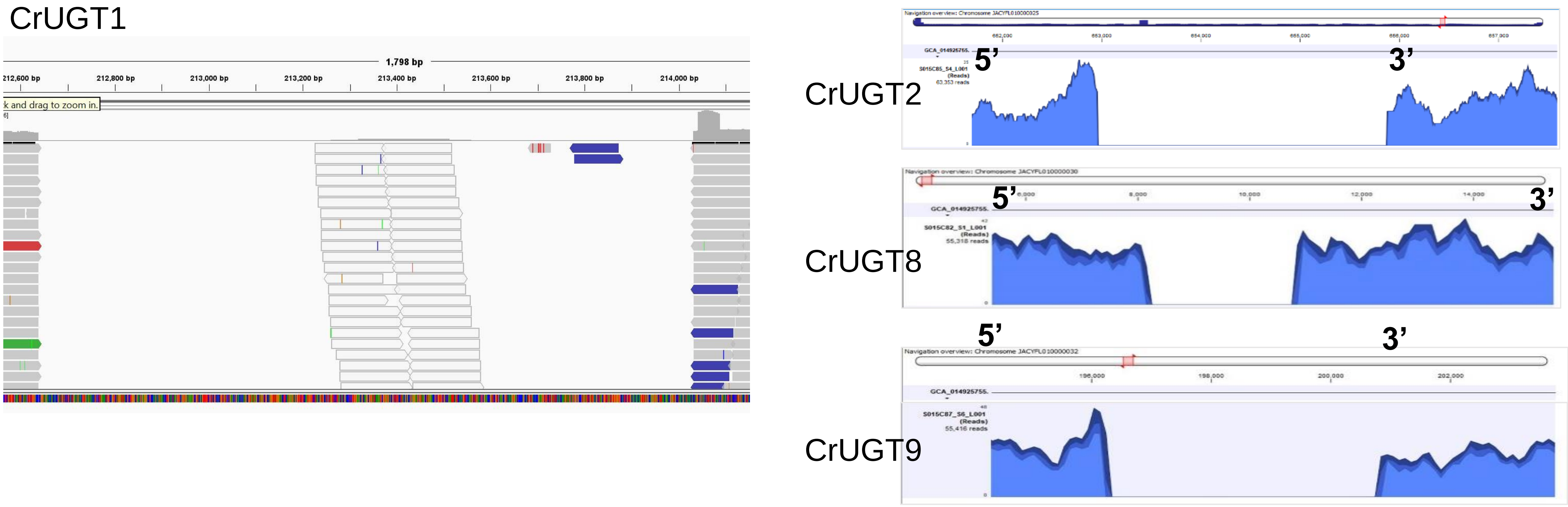

Supplemental Figure S2: Whole Genome Sequencing of selected *C. rosea* KO strains. For CrUGT1, attempts to validate by PCR were inconclusive, albeit not negative; as such this strain was sent for WGS. Screen shots showing regions covering CrUGT1 gene. Reads matching gene sequences are shown in boxes, hollow reads map quality = 0, validating replacement of CrUGT1 gene. WGS was also performed on KO strains for CrUGT2, CrUGT8 and CrUGT9. Screen shots showing regions covering the respective gene. The gap in the blue (indicting reads) indicates the gene is missing from the WGS.

Supplemental Figure S3. Overlay of the crystal structure of *Candida albicans* trehalose-6-phosphate synthase in complex with UDP-glucose (white, PDB ID # 5HUT) with the homology models of CrUTPS1 (green) and CrUTPS2 (pink). Arg 424 from CrUTPS1, Val 284 from CrUTPS2, and conformation A of UDP-glucose from the *C. Albicans* structure are shown in sticks, highlighting a potential clash between Arg 424 and UDP-glucose, with a distance of 1 Å between the closest atoms.

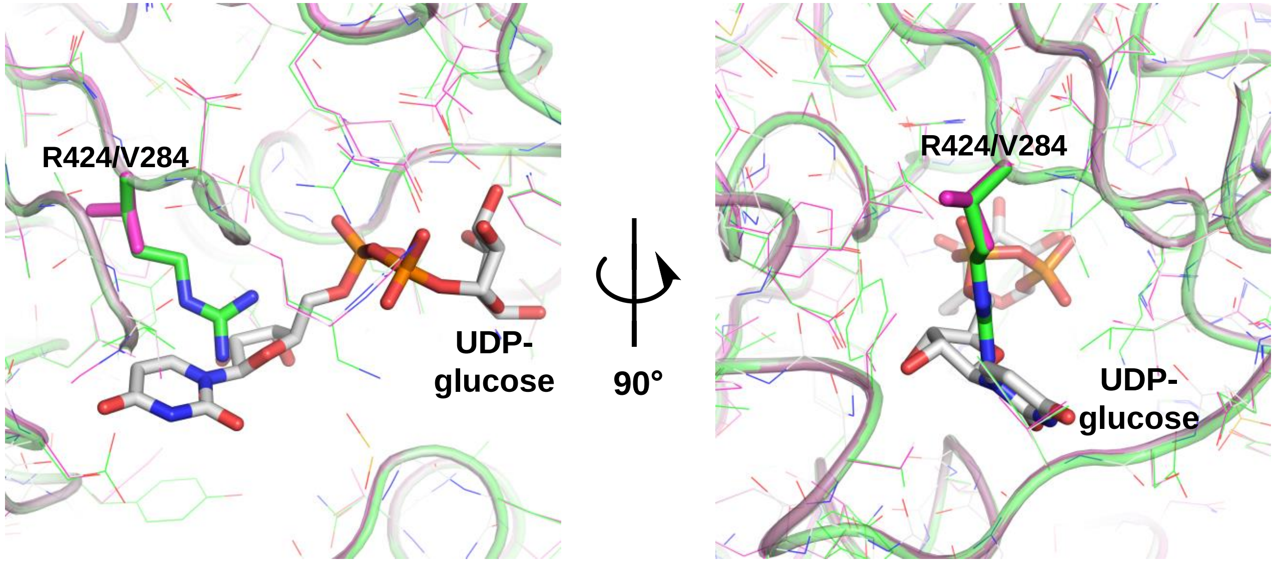

Supplement: Supplementary file 1 [file jof-09-00723-s001.zip › Supplementary File S2 (Supplementary Figures)_230605.pdf]
